# Conserved and distinct expression of circular RNAs in commercially used Marek’s disease vaccine viruses

**DOI:** 10.1099/jgv.0.002146

**Published:** 2025-09-05

**Authors:** Camille Ponsard, Alexis S. Chasseur, Pierre Lombard, Béatrice Danneels, Camille Letellier, Ahmed Kheimar, Yu You, Caroline Denesvre, Benedikt B. Kaufer, Damien Coupeau, Benoît Muylkens

**Affiliations:** 1 Namur Research Institute for Life Sciences (NARILIS), Integrated Veterinary Research Unit (URVI), University of Namur, Namur, Belgium; 2 Institute of Virology, Freie Universität Berlin, Berlin, Germany; 3 Department of Poultry Diseases, Faculty of Veterinary Medicine, Sohag University, 82524, Sohag, Egypt; 4 INRAE, UMR1282 ISP, Equipe Biologie des Virus Aviaires, Université de Tours, Nouzilly, France

**Keywords:** circular RNA, DNA packaging, herpesvirus, latency, Marek’s disease, vaccine

## Abstract

Circular RNAs (circRNAs) are covalently closed RNA molecules, supporting a wide diversity of functions. While aberrant circRNA expression stands as a recognized hallmark of cancer development, our attention has turned to investigating their role in viral infections, specifically *Mardivirus Gallidalpha 2* (GaHV-2, Marek’s disease virus) infection. In a previous study focused on the virulent GaHV-2 strain, RB-1B, we extensively catalogued circRNAs produced from virulence genes, notably from the MEQ-vIL-8 *locus* and the latency-associated transcripts (LATs) gene. Building upon this groundwork, our current investigation uncovers novel *loci* expressing viral circRNAs in distinct stages of GaHV-2 infection. Furthermore, we extend our focus to viral circRNA signatures in three commonly used Marek’s disease vaccines, the avirulent GaHV-2 (CVI988/Rispens strain), non-oncogenic *Mardivirus Gallidalpha 3* (GaHV-3) and non-oncogenic *Mardivirus Meleagridalpha 1* (MeHV-1) commercially called herpesvirus of turkey. In these vaccine viruses, we identified viral circRNA expression from a *locus* antisense to the ICP4 immediate early gene, a conserved feature across the three species. This region has been characterized herein for the first time in terms of candidate LATs’ exons and introns for GaHV-3 and MeHV-1. LATs’ circRNAs were then deeply analysed, and we observed both similarities and distinctions when compared with those of the virulent GaHV-2. Another conserved gene, encoding the DNA packaging protein, was identified as a source of circRNAs in all three species. Eventually, different levels of circRNAs were found to be expressed from the *meq locus* between virulent and avirulent GaHV-2 strains. Our findings highlight a conserved pattern of virus-derived circRNAs in these related avian alphaherpesviruses. This conservation underscores the potential significance of these transcripts in completing the viral cycle and facilitating viral spread.

## Graphical Abstract

Comparing circRNAs expressed by three Mardiviruses infecting chicken, some similarities and discrepancies were observed, both concerning which genes produce circular transcripts and at which level.

## Introduction

While the discovery of circular RNAs (circRNAs) dates back to the 1970s [1], their significance has gained prominence in the last 15 years. Initially considered as ‘potential artefacts’, circRNAs have emerged to play crucial roles in cellular processes. They are produced through an alternative splicing mechanism called backsplicing. It also uses the spliceosomal machinery, but on the opposite of U2 linear splicing during which a 5′ donor site (AG/GT) performs a nucleophilic attack on a 3′ acceptor site (YAG/G) downstream in the sequence, canonical backsplicing involves a 3′ acceptor site from an exon transcribed ahead. This process results in the generation of a circular transcript devoid of extremities [2]. In addition, U2-independent backsplicing using non-canonical splice sites was also detected, but the functional mechanism is still unknown [3,5]. At the functional level, circRNAs have been shown to be potent regulators by acting as (1) microRNA (miRNA) sponges [6,7], (2) inhibitors or enhancers of protein interactions [8], (3) scaffold triggering or regulating protein activity [9,10] and (4) RNA templates potentially translated into proteins [11,12]. These functions have also been observed in virus-derived circRNAs, particularly in herpesviruses. Notably, two circRNAs associated with immune evasion and cancer development have been identified in Epstein–Barr virus (EBV)-infected patients [13,14]. First, circBART2.2 targets the RIG-I pathway to induce immune escape by upregulating PD-L1 expression and is correlated with shorter life expectancy in nasopharyngeal carcinoma patients [10]. Second, circLMP2A is linked to poor prognosis in gastric cancer by sequestering a cellular miRNA, thereby inhibiting its regulatory effect on the TP53 pathway and promoting tumourigenesis [6].

*Mardivirus Gallidalpha 2* (GaHV-2), also known as Marek’s disease virus (MDV), causes Marek’s disease (MD). MD is characterized by immunosuppression, neurological symptoms and a rapid-onset lymphoma in infected chickens [15]. Due to its high transmission rate and pathogenicity, the entire poultry facilities can be decimated within a few weeks of initial transmission. Worldwide, MD is controlled through strict vaccination protocols. Over the years, three live-attenuated commercial vaccine strains of the *Mardivirus* genus have been used in poultry production [16]. CVI-988 strain (also named Rispens) is the most efficient vaccine and was derived from a naturally attenuated virus. This GaHV-2 strain shows a high level of identity with the virulent GaHV-2 strain, averaging 98% at the aa level for ORFs from unique regions. Besides the point mutations identified in ORFs from the repeat regions, a deletion in the latency-associated transcript (LATs) promoter sequence has also been observed [17]. The SB-1 strain from the closely related non-oncogenic GaHV-3 and the FC-126 strain from herpesvirus of turkey (HVT, *Mardivirus Meleagridalpha* 1, MeHV-1) have also been effective in preventing disease induced by virulent GaHV-2 strains [18]. These two species share an average unique ORF identity with GaHV-2 of 88.2% for GaHV-3 and 85.7% for MeHV-1. However, none provide sterilizing immunity, allowing viral replication and shedding, leading to the emergence of increasingly virulent viral strains [19]. In consequence, the MDV community is concerned about the emergence of strains capable of evading the vaccine-induced protection.

In 2022, our research group reported on the expression of GaHV-2-derived circRNAs [3]. This study identified four main *loci* of circRNA expression during lymphomagenesis: the phosphoprotein 14 kDa (pp14), a *locus* close to the origin of replication (oriLyt), the MDV EcoRI Q fragment (MEQ) *locus*, the viral telomerase RNA (vTR) *locus* and the LATs *locus*. Because all these four *loci* encode key actors for the infection, we hypothesized that circRNAs may contribute to GaHV-2 virulence, akin to miRNAs discovered in 2006 [20]. Given these observations, it was of great interest to analyse if vaccine viruses that are non-virulent would also express circRNAs. In this study, we present and characterize the circRNAs produced by different vaccine species (GaHV-3, MeHV-1) and strain (GaHV-2 CVI-988) used in the MD control programme, aiming to deepen our understanding of GaHV-2 biology and uncover potential virulence factors. Using bioinformatic tools that we established in previous studies, and molecular biology approaches, applied on the productive infection of each virus, we identified similarities and discrepancies in the circRNA profiles of the different *Mardivirus* species. First, the expression of circRNAs from the major oncogene (*meq*) was lower in the avirulent strain CVI-988 compared with the virulent RB-1B. Second, two other *loci* were detected to produce abundant circRNAs: one located in the gene encoding one DNA packaging subunit and the other localized within ‘candidates’ LATs not yet characterized in GaHV-3 and MeHV-1 species.

## Methods

### Cell lines and viruses

The mesenchymal ESCDL-1 cell line [21] was used for all viral infections reported in this study. The cells were grown in Dulbecco’s modified Eagle medium F-12 (Gibco) supplemented with 10% FBS, 1% of non-essential aa, 1 mM sodium pyruvate, penicillin (50 U ml^−1^) and streptomycin (50 µg ml^−1^).

Three viral species were used. Viruses were reconstituted from bacterial artificial chromosome systems encoding an EGFP in the mini-F cassette: GaHV-2 strain CVI-988 established by Petherbridge *et al*. [22], GaHV-3 strain SB-1 established by Petherbridge *et al*. [23] and MeHV-1 strain FC-126 established by Baigent *et al*. [24]. Viral infections were propagated by successive cell passages to reach 85% of infected cells.

### RNA extraction, library preparation and sequencing

Total RNAs were extracted from the cells infected with the corresponding viruses using TRI reagent (ThermoFisher) following the manufacturer’s protocol. Extracted RNAs were treated with 2 units of Turbo DNase (Invitrogen) for 10 µg of RNA to remove potential contaminating DNA. This step was followed by a clean-up using the RNA Clean and Concentrator 5 (Zymo Research). Five microgrammes of total RNA were sent to Novogene for sequencing (circRNA-seq or RNA-seq service). The remaining RNA was stored at −80 °C for subsequent analyses. The Novogene’s protocol for circRNA-seq includes a circRNA-enriched procedure (ribo-depletion and RNase R-treatment) before the library preparation and sequencing. The obtained sequencing data were next analysed using vCircTrappist [25]. Sequences were aligned along respective genomes: GaHV-2 CVI-988 (Acc. n° DQ530348), GaHV-3 SB-1 (Acc. n° NC_002577) and MeHV-1 FC-126 (Acc. n° NC_002641). External repeats were removed for circRNA analyses as described in vCircTrappist advice. In addition, one sequence within the glycoprotein C gene was not covered in our analysis, potentially introducing artefacts during the sorting of spliced reads. In consequence, we decided to remove these bases for further analysis:

- GaHV-2 CVI-988 (Acc. n° DQ530348 without bases 103681–103781)
- GaHV-3 SB-1 (Acc. n° NC_002577 without bases 99640–99740)
- MeHV-1 FC-126 (Acc. n° NC_002641 without bases 93872–93972)

Concerning the analysis of linear mRNA-seq, the forward-spliced reads were extracted using the first step of the vCircTrappist pipeline and analysed through a new home-made script available on GitHub (https://github.com/achasseu/CircRNA_MDV_Vaccines). Concisely, a Python script checks and orients misstranded reads based on common and/or canonical splicing patterns. Then, the reads are visualized using an R script.

### Reverse transcription and PCR

Four microgrammes of RNA were reverse transcribed following the protocol of SuperScript IV enzyme (ThermoFisher). RNAs were heated with random hexamers (50 µM) at 95 °C for 5 min, instead of the 65 °C indicated in the original protocol, to overcome the strong secondary structures of circRNAs. One microlitre of the reverse transcription (RT) product was used for the subsequent PCR. For the circular PCR amplifications, we used 5 units of GoTaq G2 polymerase (Promega) and followed the manufacturer’s instructions. Linear LATs amplifications were realized with 2.5 units of TaKaRa LA Taq for GaHV-3 and 1 unit of Q5® High-Fidelity DNA Polymerase (NEB) for MeHV-1. The 3′ RACE PCRs were conducted following the manufacturer’s protocol of the GeneRacer™ Kit (ThermoFisher). Primers used are presented in Supplementary Material 1. Migrations were realized in 1% agarose gel with Midori Green (Nippon Genetics) during 75 min at 100 V. Ladders used are either SmartLadder (SL) (Promega) or SmartLadder SF (SF) from Eurogentec. pGEM®-T Easy Vector Systems were chosen to clone PCR products and be able to sequence them with the Mix2Seq Service of Eurofins Genomics. Alignments were then realized on Geneious software (Biomatters).

### Evaluation of similarities between proteins of different viruses through bioinformatic analysis

aa sequences were submitted to blastp against the *Herpesvirales* database (taxid: 548681) in UniProtKB/SwissProt, or in non-redundant proteins for *Mardivirus*. Pairwise alignments were used to calculate the percentage of similarity between each GaHV-2 protein and their homologues from two other Mardiviruses (GaHV-3 SB-1 and MeHV-1 FC-126) and (when present) human members of alpha- (HHV-1, -2 and -3), beta- (HHV-5, -6 and -7) and gamma- (HHV-4 and -8) herpesvirus subfamilies. The similarity percentage for each subfamily was determined by multiplying the average query coverage (the proportion of the GaHV-2 sequence aligned, accounting for differences in length) by the average percentage of identity in the aligned regions.

## Results

### GaHV-2 CVI-988 strain and related *Mardivirus* species used in MD vaccine programme express circRNAs

In our previous study, we described circRNAs from a large panel of samples infected with the RB-1B oncogenic GaHV-2 strain [3]. Hot spots of virus-derived circRNAs were observed, notably from *meq* and LATs transcriptional units. While the meq *locus* is unique to GaHV-2 and inherent to its tumourigenesis, the LATs gene is a hallmark of *alphaherpesvirus*. However, it has not been characterized yet in GaHV-3 and MeHV-1. In this study, we investigated the expression of circRNAs in live attenuated MD vaccines, namely, the CVI-988 avirulent GaHV-2 strain, and two additional *Mardiviruses*, GaHV-3 and MeHV-1 (HVT).

RNAs were isolated from productive lytic *in vitro* infections with each of the three viruses and submitted to RNA sequencing after circRNA enrichment. Viral reads were analysed to detect vaccine-derived circRNAs using our recently developed bioinformatic pipeline [25]. RNA-seq data were sorted by focusing on splicing events and more specifically on those showing backsplicing signatures. In the three infections, reads displaying discontinuous mappings, reflecting splicing, represented 3–7% of the viral sequence data (Table 1). Among the spliced transcripts, numerous showed exons in scrambled order, suggesting that they originated from circularization events where acceptor splice sites are localized upstream of the donor sites. These transcripts will be referred to as ‘back-splice junction reads’, as the ‘back-splice junction’ (BSJ) sequence is built through the assembly of the two splice sites involved in circRNA biogenesis. They accounted for 30% of the spliced transcripts in GaHV-2 CVI-988 and MeHV-1 and 17% in GaHV-3. Altogether, virus-derived back-splice junction reads represented 1.0, 0.6 and 2.0% of the viral transcriptomic datasets gathered from attenuated GaHV-2 CVI-988, GaHV-3 and MeHV-1, respectively (Table 1).

**Table 1.** Analysis of circRNA reads in species used in the MD vaccination programme

Species-strain-pathotype	% identity to RB-1B (a)	RNA-seq data analysis		
		**% of spliced reads (b** **)**	**% of BSJ reads (c** **)**	**% of canonical BSJ reads (d** **)**
GaHV-2/CVI-988/a	98	3.5	1	10.9
GaHV-3/SB-1/a	88.2	3.7	0.6	16.8
MeHV-1 (HVT)/FC-126/a	85.7	7	2	30.2

a, avirulent; (a) % Identity with RB-1B at amino acid level for ORF from the unique regions; (b) % spliced viral reads in viral reads; (c) % back-splice junction (BSJ) reads in viral reads; (d) % canonical back-splice junction reads in back-splice junction reads.

### CircRNA patterns show discrepancies and similarities between virulent and vaccine viruses

**Fig. 1.**
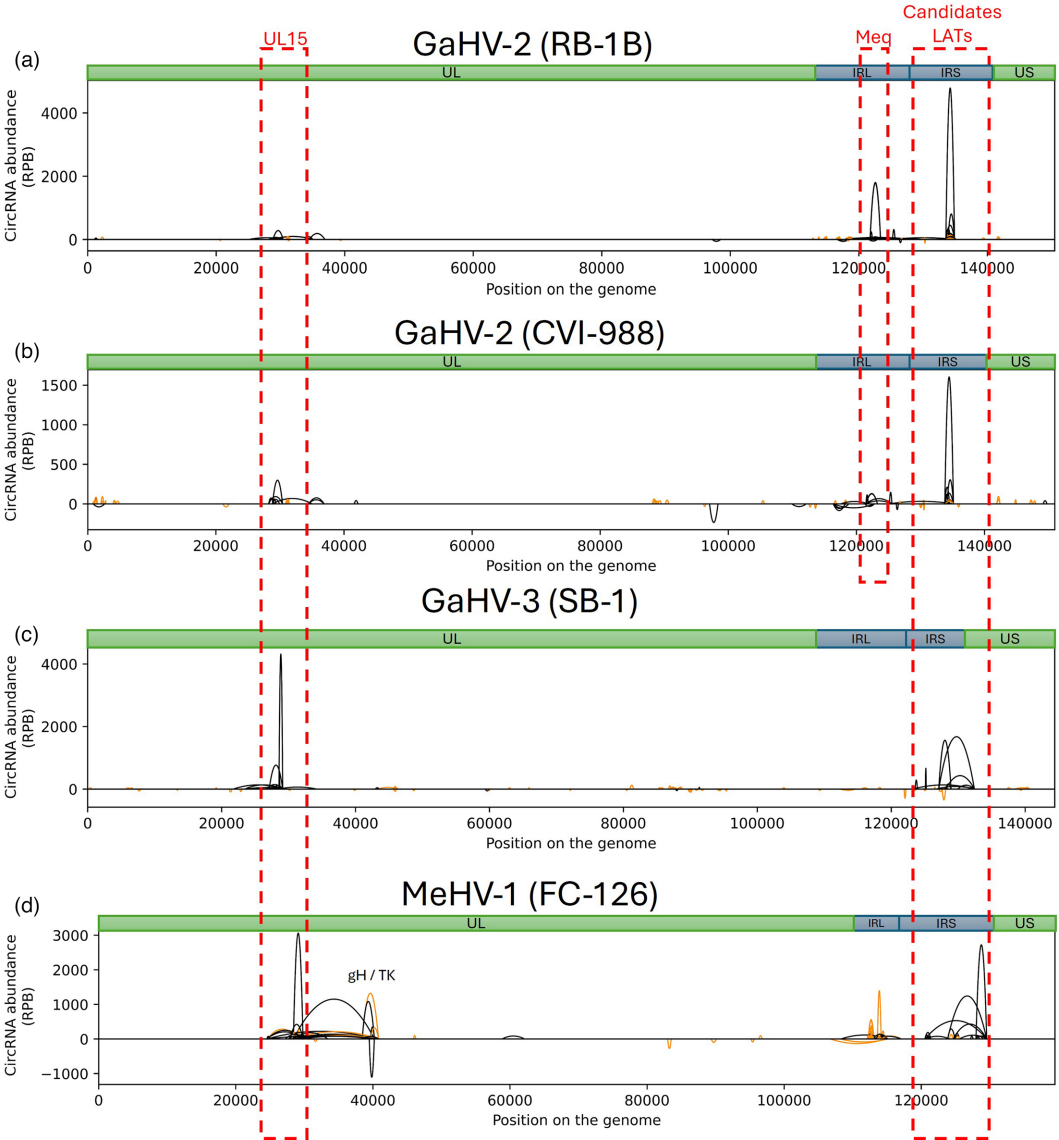
The 100 most represented circRNAs produced by GaHV-2 RB-1B (a), GaHV-2 CVI-988 (b), GaHV-3 (c) and MeHV-1 (d). Each parabola represents a single circRNA. The height of parabolas on the Y-axis represents the normalized abundance of each individual circRNA [expressed in back-splice junction reads per billion (RPB) reads mapped on the viral genome], and negative values indicate a mapping on the antisense strand of the viral genome. The X-axis represents the position on the viral genome devoid of its terminal repeats. Unique long (UL) and unique short (US) regions are depicted in green. Internal repeat long (IRL) and internal repeat short (IRS) regions are depicted in blue. External repeats were removed from the genome for convenience in the analysis. The orange colour depicts the circRNAs produced through non-canonical splicing [U2-independent using splice sites different from (GU-AG)]. The black colour depicts the circRNAs produced through U2 canonical splicing.

Back-splice junction reads were mapped onto the corresponding viral genomes. CircRNA expression profiles of the three vaccine species were compared with the one previously established for GaHV-2 RB-1B virulent strain (Fig. 1). To this end, sequences were processed to extract the 100 most frequent BSJs. The circRNAs derived from these BSJ were represented in the shape of parabolas on the genome, where the extremities highlight the positions of the two splice sites involved in backsplicing, and the height of the peak indicates the abundance of each BSJ.

First, the CVI-988 circRNA profile was compared with the one of RB-1B, a very virulent strain, since they both belong to the GaHV-2 species. Accordingly, CVI-988 also encodes GaHV-2-related oncogenes such as *meq*. Circular transcripts were already described from the meq *locus* in RB-1B [3]. These circMeq transcripts partially encompass the coding region of the *meq* gene but are not restricted to it, as some variants also include sequences derived from the *vIL-8 locus*. (Fig. S1, supplementary figures are available in Supplementary Material 2). The same circRNA isoforms were observed in CVI-988 and RB-1B, but they were barely detected in the avirulent strain. While the *meq* gene is a hot spot of viral circRNA production in the virulent strain, the number of circMeq reads is reduced by a factor of 20 in the vaccine CVI-988 strain (Fig. 1a, b).

Next, two *loci* were detected to produce circRNAs in the three Mardiviruses analysed: UL15, encoding the DNA packaging unit, and the *locus* antisense to ICP4, corresponding to LATs in GaHV-2, even though LATs have not previously been described for GaHV-3 and MeHV-1 (Fig. 1). For brevity, we will refer to these circRNAs as circUL15s and circLATs in subsequent paragraphs. The level of circUL15s expression is similar in the two GaHV-2 strains, with ~300 reads per billion in both, while higher for GaHV-3 and MeHV-1. The presence of circUL15s and circLATs in three closely related alphaherpesviruses suggests a possible biological relevance of these circRNAs in the viral cycle. While the production of circRNAs from these *loci* is conserved across the different viruses, the splice sites are not similar at the nt level. Additionally, the three species exhibit differences in the number of alternative BSJs for the same *locus*, resulting in either a predominant circRNA or a collection of alternative ones (Fig. 1).

Besides these three *loci* (meq, UL15 and LATs) from which the most abundant virus-derived circRNAs were detected, a comprehensive view of all back-splice junction reads, available in Fig. S2 (available in Supplementary Material 2), revealed a high diversity of minor BSJ reads that did not result from U2-dependent splicing machinery. They are represented by the orange coverage signal. They were identified at several *loci* such as the glycoprotein C (gC), the vTR, the viral lipase (vLIP) and lytic genes surrounding the DNA origin of replication (oriLyt). In addition, vLIP and gC circRNA levels were increased in CVI-988 compared with RB-1B. To date, the precise mechanism underlying the production of non-canonical circRNAs remains unclear. It has been observed, however, that these circRNAs lack the typical U2-type splice site motifs (GU-AG) at the sites of circularization.

Taken together, these results highlight two *loci* that express circRNAs across three viral species used intensively in MD vaccine programmes: UL15 and LATs. However, while these circRNA-seq results describe backsplicing events, they do not provide a complete picture of integral circRNA transcripts. To obtain full circRNA sequences, including all alternative internal splicing events, an RT-PCR approach followed by cloning and Sanger sequencing was necessary and was subsequently employed. This is particularly important in the case of multiply spliced transcripts, such as the LATs.

### Identification of circLATs antisense to ICP4 gene from the three vaccine species

**Fig. 2.**
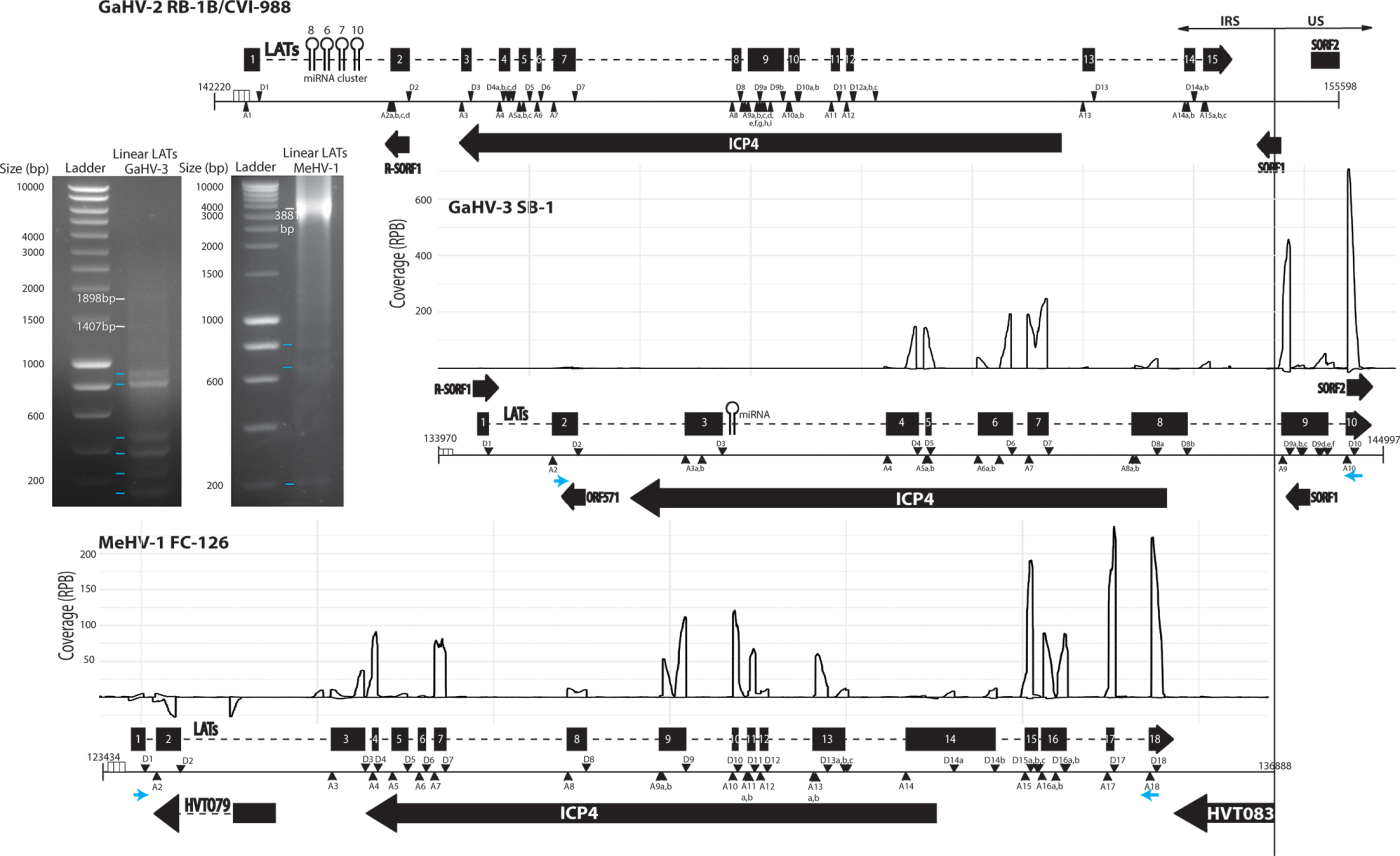
Maps of LATs locus in GaHV-2, GaHV-3 and MeHV-1. Genes are represented by thick black arrows with boxes separated by dotted lines if different exons are present for one gene. Black triangles and inverted black triangles represent the U2 acceptor splice site (A) and U2 donor splice site (D) of LATs. Sequences and positions of splice sites are available in the online Supplementary Material 1. miRNAs are shown by hairpins while small blue arrows indicate the positions of primer used for linear PCR amplifications (Supplementary Material 1). The migrations of PCR amplicons are shown on the left side, with major signals highlighted by small blue lines. Strand-specific RNA sequencing coverage plots for GaHV-3 and MeHV-1 were generated exclusively from reads spanning exon–exon junctions. Coverage is expressed in reads per billion (RPB) reads mapped to the viral genome. Positive values represent sense strand reads, while negative values indicate antisense coverage derived from the negative DNA strand. Sorting of these spliced reads was performed using a new home-made script available on GitHub (https://github.com/achasseu/CircRNA_MDV_Vaccines).

Since LATs have not been previously described in GaHV-3 and MeHV-1, the initial objective of our study was to identify and characterize LATs in these species and characterize their features. LATs of GaHV-2 have been described over the years, starting with an initial description of a 10 kb RNA in 1994 [26,27] to a complete map of 15 exons in 2012 by Strassheim *et al*. [28]. No ORF has been identified, confirming their classification as long non-coding RNAs. In addition, they serve as precursors for four miRNAs. They are localized antisense to the ICP4 immediate early gene. In consequence, to identify LATs exons in the two other Mardiviruses, we combined different techniques to study the *locus* antisense to ICP4. First, linear RNA-seq data were sorted by a module of the vCircTrappist pipeline to keep only reads spanning exon–exon junctions and generate coverage results (Fig. 2). 3′ RACE PCR (rapid amplification of cDNA ends by PCR) was done to determine transcript 3′ ends (Fig. S3, available in Supplementary Material 2). These results were further validated and refined by PCR amplification using primers designed to target the two terminal predicted exons. Amplicons were visualized following migration through an agarose gel (Fig. 2) and then cloned to be able to identify the diversity of transcripts by Sanger sequencing. Being able to obtain amplicons using primers that are so far apart demonstrates that all the exons we identified originate from a common initial transcript. The sizes of the bands we obtained were consistent with the expected transcript lengths. For GaHV-3, excluding exons 3 and 8 – which appear to be less frequently included – yields amplicons of either 1,407 or 1,898 bp, depending on the alternative splice site used for exon 9. In the case of MeHV-1, the cumulative length of all exons results in a 3,881 bp amplicon (Fig. 2). Then, other lower bands were observed for both viruses, highlighting alternative internal splicing with exons skipping. By integrating all these data, we generated the first LATs maps for GaHV-3 and MeHV-1, presented alongside the GaHV-2 LATs map previously described (Fig. 2). Our analysis predicted 11 exons for GaHV-3 and 18 exons for MeHV-1, each with multiple alternative splice sites. Notably, both the exons and their corresponding splice sites appear to be species-specific. Interestingly, unlike GaHV-2 and MeHV-1 LATs, GaHV-3 LATs are not confined to the short repeat (RS) region of the genome as two exons and their two alternative polyadenylation sites are located in the unique short (US) region.

**Fig. 3.**
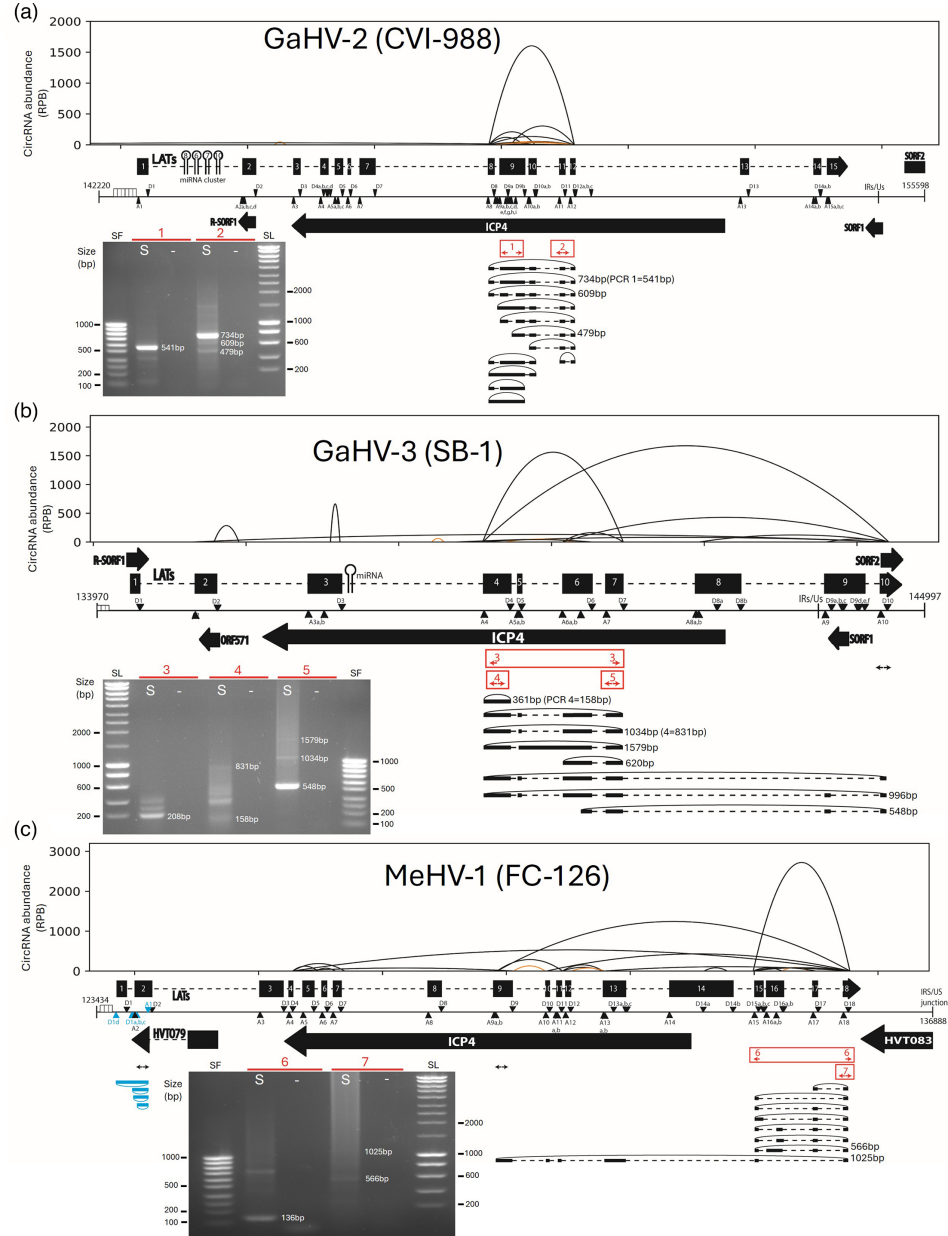
Mapping of the LATs-derived circRNAs (circLATs) during a GaHV-2 CVI-988 (a), GaHV-3 (b) and MeHV-1 infection (c). For each virus, circRNAs identified from circRNA-seq are above the genomes, while PCR sequencing data are represented below with isoforms depicted and migrations of amplicons on agarose gels. Major BSJs identified with vCircTrappist are represented in the shape of parabolas. The Y-axis represents the normalized abundance of each individual BSJ [back-splice junction containing reads per billion (RPB) reads mapped on the viral genome]. The X-axis represents the position on the viral genome devoid of its terminal repeats. The orange colour depicts non-canonical back-splicing [U2-independent using splice sites different from (GU-AG)]. In the middle, a genome map is shown with genes represented by thick black arrows or boxes separated by dotted lines if different exons are present for one gene. Black/blue triangles and inverted black/blue triangles represent the U2 acceptor splice site (A) and U2 donor splice site (D), respectively, in sense and antisense. Sequences and positions of splice sites are available in the online Supplementary Material 1. On the bottom, PCR sequencing results present which exons were precisely identified in the different circRNAs for each BSJ. CircRNAs are depicted by black bold lines in sense or blue in antisense. They are bound by dotted lines, representing the introns. The positions of divergent primer couples are represented by red double arrows with some of them numbered when migrations are shown (Supp Material 1). ‘S’ lanes show amplicons generated from cDNA derived from infected cells, while ‘–’ lanes represent negative controls using water.

With these novel maps, we were able to better analyse circRNA sequences. Fig. 3 summarizes all the data obtained regarding circLATs, displaying BSJ identified from high-throughput and Sanger sequencing data in parallel, as well as PCR products resolved on gel. The first important observation is the clear concordance between the results of the two methods, showing that vCircTrappist is a reliable tool to identify viral circRNAs. This reliability applies not only to global circRNA profiling but also to the precise identification of circular transcripts from specific *loci*. Visualization of PCR products enables the identification of major and minor transcript variants, based on the relative intensity of each band and their correspondence to the predicted sizes of isoforms identified by sequencing (Fig. 3).

Interestingly, while circLATs are produced by all three virus species, they are not generated from the same positions in the LATs *locus*. Specifically, although all circLATs derived from the strand opposed to ICP4, their proximity to the 5′ end of ICP4 varies among species. In GaHV-3, as in GaHV-2, circLATs include mainly exons localized antisense to the middle part of ICP4 (Fig. 3). In addition, GaHV-3 also produces circular transcripts involving additional exons found in the US region (Fig. 3). In contrast, in MeHV-1, apart from a higher diversity of circLATs, the predominant splice sites are located upstream of the ICP4 coding sequence (Fig. 3). While this suggests that they may not be directly related to ICP4, it remains possible that they are localized in its 5′ UTR or its promoter. Additionally, some transcripts include exons directly antisense to ICP4, extending close to its STOP codon according to circRNA-seq results.

### Exon 2 of the highly conserved UL15 gene expresses circRNAs in all three viruses

The second *locus* consistently expressing circRNAs in the three vaccine viruses is found in the gene encoding the UL15 protein, a DNA packaging terminase subunit. This gene is present in all members of the *Orthoherpesviridae* family. To assess its conservation compared with other key viral proteins, the aa sequences of various GaHV-2 proteins were submitted to blastp against the Herpesvirales (taxid: 548681) database [29]. This analysis aimed at generating pairwise alignments with corresponding proteins from human herpesviruses in the alpha (HHV-1, -2 and -3), beta (HHV-5, -6 and -7) and gamma (HHV-4 and -8) subfamilies, provided that they were present in the database. Based on these results, we quantified the homology between GaHV-2 proteins and their homologues (Table 2). The high similarity observed for pUL15 demonstrates its strong conservation within the *Orthoherpesviridae* family. Specifically, the percentages for pUL15 are higher or comparable to those of two proteins conserved among herpesviruses: the DNA polymerase and the envelope glycoprotein B. Furthermore, pUL15 shows higher similarity than other proteins involved in the encapsidation process (Table 2) [30,31].

**Table 2.** Similarity percentage between GaHV-2 proteins and their homologues (when present) from two other Mardiviruses and human members of alpha- (HHV-1, -2 and -3), beta- (HHV-5, -6 and -7) and gamma- (HHV-4 and -8) herpesvirus subfamily

	Protein compared	Chicken *Mardivirus* (GaHV-3/MeHV-1)	Human *Alphaherpesvirinae* (HHV1-2-3)	Human *Betaherpesvirinae* (HHV5-6-7)	Human *Gammaherpesvirinae* (HHV4/8)
Proteins involved in DNA encapsidation	**UL15**	**76**	**54**	**28**	**25**
	UL28	69	43	22	18
	UL33	63	40	14 (HHV-5)	/
	UL6 (portal)	62	37	18	19
Envelope proteins	**gB**	**81**	**48**	**25**	**24**
	gL	49	15 (HHV-3)	/	/
Early proteins	**DNA pol**	**72**	**50**	**26**	**31**
	TK	63	25	/	/
Immediate early proteins	ICP27	53	16	/	/
	ICP4	27	9	/	/

The way to calculate percentages of similarity is described in the ‘Methods’ section.

DNA pol, DNA polymerase; gB, glycoprotein B; gL, glycoprotein L; TK, thymidine kinase.

The majority of current functional knowledge on pUL15 was obtained from HHV-1 studies [32,33]. Two exons are defined as encoding: first, a ‘motor’ domain that binds ATP via its Walker A/B motifs to translocate DNA into capsids during packaging, and second, a ‘nuclease’ domain that cleaves concatemeric DNA [34]. As the pairwise alignment of pUL15 from GaHV-2 and HHV-1 shows 54% identity (Table 2), and we detected conservation of functional elements, such as the two Walker boxes (Fig. S4, available in Supplementary Material 2), we can conclude that characteristics of HHV-1’s UL15 can be extrapolated to GaHV-2 protein.

**Fig. 4.**
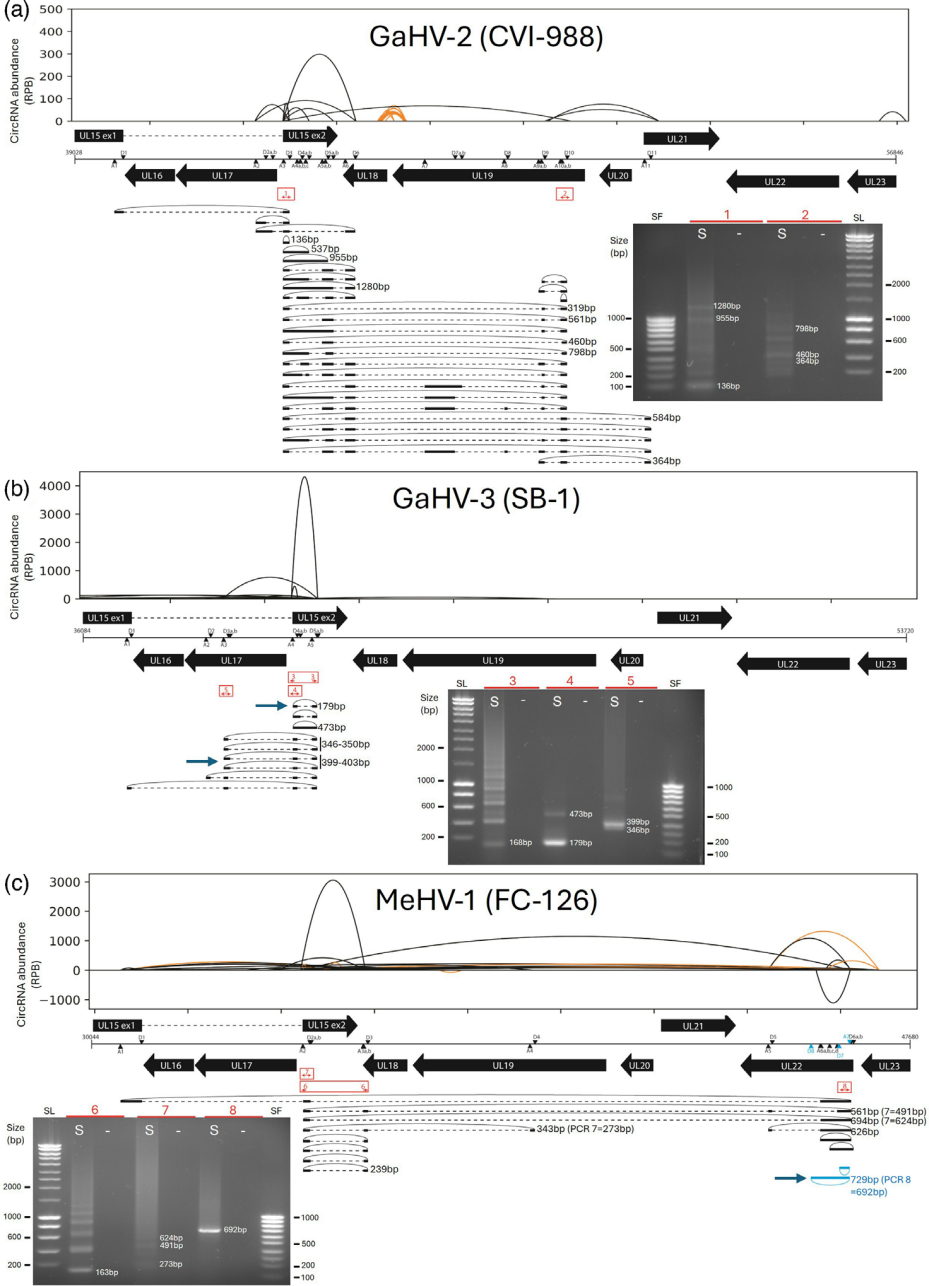
Mapping of the UL15-derived circRNAs (circUL15s) during a GaHV-2 CVI-988 (a), GaHV-3 (b) and MeHV-1 infection (c). For each virus, circRNAs identified from circRNA-seq are above the genomes, while PCR sequencing data are represented below with isoforms depicted and migrations of amplicons on agarose gels. One hundred major BSJs identified with vCircTrappist are represented in the shape of parabolas. The Y-axis represents the normalized abundance of each individual BSJ [back-splice junction containing reads per billion reads (RPB) mapped on the viral genome], and negative values indicate a mapping on the antisense strand of the viral genome. The X-axis represents the position on the viral genome devoid of its terminal repeats. The orange colour depicts non-canonical back-splicing [U2-independent using splice sites different from (GU-AG)]. In the middle, a genome map is shown with genes represented by black thick arrows or boxes separated by dotted lines if different exons are present for one gene. Black/blue triangles and inverted black/blue triangles represent the U2 acceptor splice site (A) and U2 donor splice site (D), respectively, in sense and antisense. Sequences and positions of splice sites are available in the online Supplementary Material 1. On the bottom, PCR sequencing results present which exons were precisely identified in the different circRNAs for each BSJ. CircRNAs are depicted by black bold lines in sense or blue in antisense. They are bound by dotted lines, representing the introns. The positions of divergent primers are represented by red double arrows with some of them numbered when migrations are shown (Supp Material 1). ‘S’ lanes show amplicons generated from cDNA derived from infected cells, while ‘–’ lanes represent negative controls using water.

The expression of circRNAs from the conserved UL15 *locus* was analysed and is presented in Fig. 4, with results from PCR and both high-throughput and Sanger sequencing shown in parallel. The lengths of the amplicons detected with high read per billion counts in the circRNA-seq quantification largely matched the band sizes observed on the gel accounting for the distance between primers (Fig. 4). When comparing the two techniques, it becomes clear that one parabola reflects only the BSJ sites and therefore corresponds to multiple isoforms that differ in internal splicing, resulting in length variation. Upon analysis of these major isoforms, it appeared that, for each Mardivirus, a predominant circRNA was localized within exon 2 of UL15 (Fig. 4). Besides this one, additional alternative circularization splice sites were found either in the described intron of UL15 or further downstream on the positive strand. These exons, localized in sense but after the STOP codon of UL15, are likely associated with the 3′ UTR of UL15. In addition, one exon was found to map to the UL21 sequence in CVI-988 (Fig. 4). In MeHV-1, two circular transcripts were also detected originating from the *UL22* region on the negative strand. The size of this longer antisense transcript corresponds to the 692 bp brighter band observed on the gel with PCR 8.

## Discussion and conclusion

GaHV-2 is a herpesvirus that causes lethal lymphomas in chickens. Its virulence is attributed to multiple factors ranging from the MEQ oncoprotein to non-coding RNAs (e.g. MDV-miR-M4 and vTR). We recently described GaHV-2-derived circRNAs in various infection models and associated them with virulence genes in lymphoma-derived samples, notably spleens collected from infected chickens [3]. Following this initial work, we investigated the potential link between circRNA expression and GaHV-2 virulence. In this study, we decipher MD vaccine-derived circRNAs. Comparing their circRNA profile to the one of the virulent RB-1B strain, differences and similarities were highlighted. First, CVI-988 expresses 20 times less circRNA from the meq *locus* compared with RB-1B. Then, two specific *loci* were identified to produce circRNAs in three related *Mardivirus* species. Particularly, we identified circRNAs in UL15, the DNA-packaging unit, and in the LATs *locus*. Interestingly, while the expression spots were conserved, we did not observe sequence conservation of the splice sites of these *loci*. Nevertheless, these two genes are shared by numerous herpesviruses as the majority of alphaherpesviruses seem to produce linearly spliced LATs [35,37], and we confirmed here the global conservation of UL15 protein in the *Orthoherpesviridae* family. Our results regarding pUL15 conservation corroborate with the study of Brito and Pinney, in which they analysed protein domains in *Orthoherpesviridae*. They demonstrated that the 2 pUL15 domains (DNA packaging C- and N-terminal) are among the 28 encoded by all members of the family in a set of 274 non-redundant herpesvirus domains [31]. In consequence, detecting circRNAs encoding partly the sequence of these genes in three different *Mardivirus* species indicates that they may play a significant role in the viral cycle, paving the way for potential new attenuation strategies.

Due to its definition as ‘the major oncogene of the virus’, *meq* is probably one of the most studied genes of GaHV-2 [38]. In this context, the relation between *meq* variation and virulence of the strain is of great interest. Conflicting data were previously reported about the implication of short and long isoforms of the MEQ transcriptional factor in GaHV-2 virulence. On one hand, very virulent (vv) and very virulent plus (vv+) GaHV-2 isolates express MEQ isoform with a low number of proline-rich repeats (PRRs), while attenuated ones express longer isoforms (L-MEQ) (Fig. S1B, available in Supplementary Material 2) [39,40]. On the other hand, it was discovered that the long isoform with additional PRRs is active, unlike what could be expected for an avirulent strain [41]. Indeed, characterization of its functionality highlighted an increased trans-repression activity accompanied by a decreased transactivation activity. In addition, it was more recently shown that replacement of the canonical MEQ isoform in a virulent GaHV-2 strain with the long isoform obtained from the attenuated GaHV-2 CVI-988 strain strikingly increased the pathogenesis of the recombinant virus, proving again the activity of this long isoform [42]. Given the transcriptional factor activity of L-MEQ, it could be hypothesized that when it is introduced into a virulent strain, it interacts with target promoter(s) absent in CVI-988 to induce pathogenesis. Our observation that CVI-988 expresses 20 times less circMeq compared with the virulent RB-1B strain adds a layer to these variable profiles between pathotypes. Given that circMeq originates from the same promoter as the linear meq transcripts, it would be informative to investigate the levels of linear meq in parallel. Since circRNA-seq samples were RNAse R treated before sequencing, linear transcript expression information was lost. Thus, additional RT-qPCR assays should be carried out using untreated RNA samples to assess simultaneously circular versus linear meq transcripts. This would help determine whether changes in circMeq expression are due to alterations in general promoter activity or are the result of specific splicing regulations. If the latter is the case, sequence variations could be analysed to identify potential *cis*- and *trans*-acting splicing factors that influence the balance between linear and circular meq splicing.

In GaHV-2, while LATs’ functions are not characterized yet [26,43], they were shown to be essential to virulence. Indeed, a deletion of their promoter sequence leads to a complete attenuation of the virus (under patent WO2014125369A3 [44]). In consequence, finding LATs expressed in CVI-988 and confirming the presence of multi-exon transcripts antisense to ICP4, corresponding in all likelihood to LATs, in avirulent GaHV-3 and MeHV-1, could be surprising. One hypothesis to explain the presence of LATs in avirulent strains could be that a basal level of LATs is required in the establishment and/or maintenance of their latency. Then, during this established latency, other oncogenes are expressed by virulent strains, leading to lymphomas, whereas oncogenes are absent in avirulent strains. Negative correlation between LATs and immediate early gene transcribed antisense to them; ICP0 in HHV-1 and ORF61 in HHV-3 have already been described [36,45], suggesting interaction with ICP4 in GaHV-2. In consequence, finding circRNAs composed of the sequence antisense to ICP4 or its promoter in several viruses could indicate that one part of LATs roles can be supported by circLATs. Recently, Yang and his collaborators also demonstrated the presence of circRNAs from LATs in HHV-3 (*Varicellovirus humanalpha 3*) and showed that modification in the level of these peculiar transcripts has an impact on viral resistance to acyclovir [46]. CircRNAs from LATs were also identified in HHV-1 infection [5]. Given that circularization of transcripts promotes self-interactions and double-stranded RNA structures, we propose that the role of circLATs might be associated with conserved or host-adapted structural motifs across different viral species. This could be illustrated by circBART2.2 in EBV. The BamHI A rightward transcripts (BART) are long non-coding RNAs expressed during the EBV latency and associated with oncogenesis. It was described that one of the derived circRNAs – circBART2.2 – interacts with the RIG-1 protein, thereby promoting tumour immune escape [10].

The discovery of several circUL15s in three closely related alphaherpesviruses indicates that they might have a functional role. This first description is the starting point of larger studies to better understand their production and identify their functions. Indeed, it remains unclear whether all circUL15 exons originate from common transcripts spanning from the beginning of UL15 to the 3′ UTR of UL21, suggesting the presence of a polycistron, or from different transcripts combined through trans-splicing [47]. Hypotheses concerning their roles are either interaction with antisense neighbouring gene transcripts such as UL17-18-19-22, or encoding an alternative nuclease that could have a role apart from DNA packaging. It was notably described in HHV-1 that UL15 encodes a second shorter protein called UL15.5 through an alternative START site localized in exon 2 that was not required for viral replication in cell culture (Fig. S4, available in Supplementary Material 2) [32,33].

In conclusion, while our work initially aimed at identifying new GaHV-2 virulence factors specific to virulent strains, we identified conserved circRNAs in different related alphaherpesviruses. These circRNAs might play a role in the viral cycle and thus deserve further characterization of their functions. To achieve that, future studies will aim to silence major circRNAs of LATs and UL15 to investigate their role in the viral cycle, latency and vaccine efficiency. GaHV-2-related species figure as good models to start investigations of these circRNAs given the fact that the circRNA hotspots are located on *loci* conserved among the *Alphaherpesvirinae* subfamily and that *in vivo* experiences with mutant virus are possible in their natural host.

## Supplementary material

10.1099/jgv.0.002146 Supplementary Material 1.

10.1099/jgv.0.002146 Supplementary Material 2.

## Abbreviations

BSJ: back-splice junction
circRNA: circular RNA
EBV: Epstein–Barr virus
HVT: herpesvirus of turkey
LAT: latency-associated transcript
MD: Marek’s disease
MDV: Marek’s disease virus
miRNA: microRNA
PRRs: proline-rich repeats
RT: reverse transcription
vTR: viral telomerase RNA
