# Supplementary material for: Conserved and distinct expression of circular RNAs in commercially used Marek’s disease vaccine viruses
Source: J Gen Virol. 2025 Sep 5;106(9):002146. doi: 10.1099/jgv.0.002146 (PMC12451640; doi:10.1099/jgv.0.002146)
Supplement: Supplementary Material 2. [file jgv-106-02146-s002.pdf]

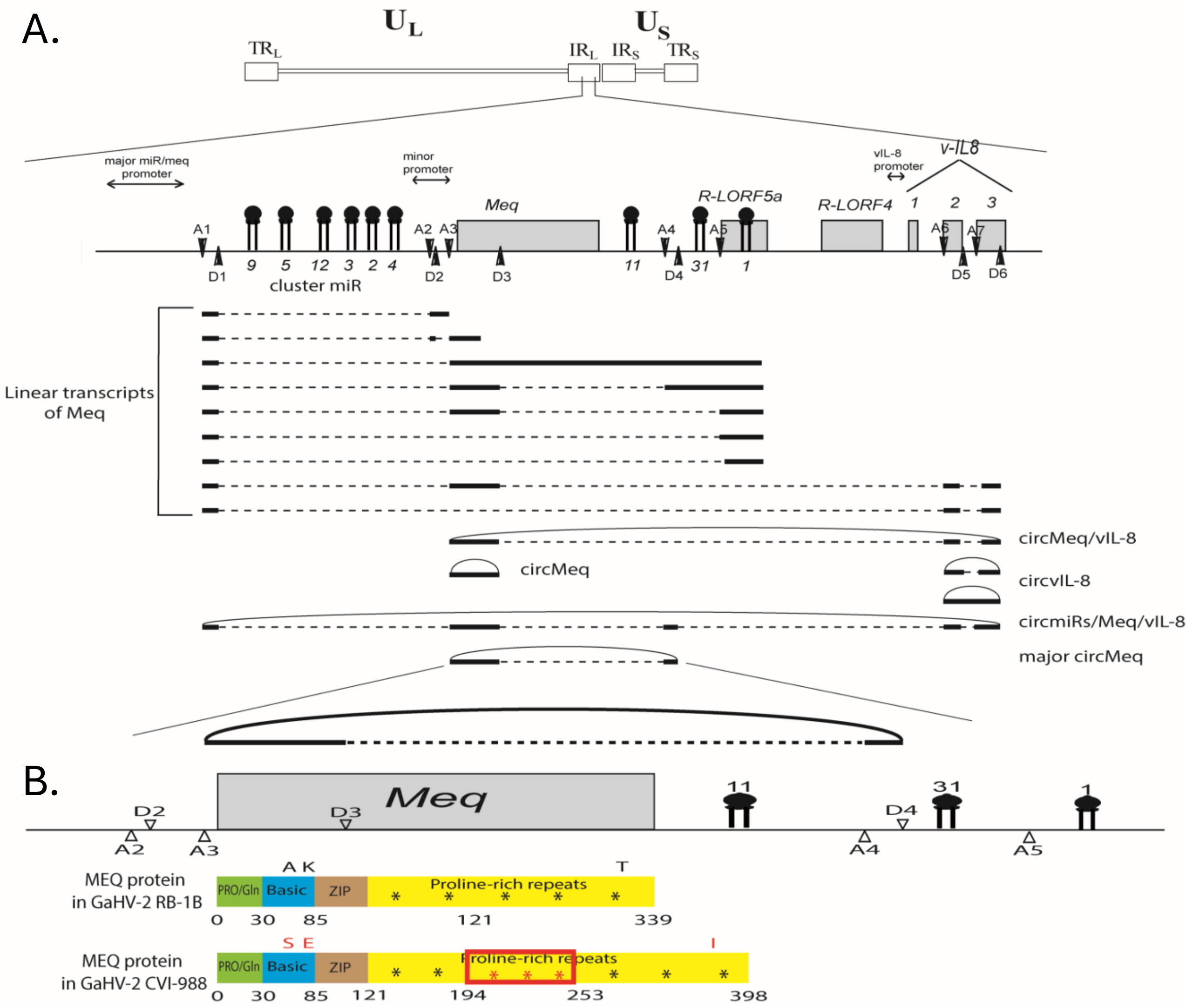

Figure S1 : **meq locus with its linear and circular transcripts diversity in parallel to protein domains in GaHV-2 RB-1B and CVI-988. A.** Transcripts derived from meq locus. GaHV-2 genome is shown on the top with terminal/internal repeats long (TRL/IRL), unique long (UL), terminal/internal repeats short (TRS/IRS) and unique long (US) regions. Grey boxes represent the proteins, hairpins the microRNAs and triangles/inverted triangles are respectively U2 acceptor splice site (A) and U2 donor splice site (D). Linear and circular transcripts are depicted black bold bound by dotted lines, representing the introns. B. MEQ domains in virulent and avirulent strains of GaHV-2; proline/glutamine (Pro/Gln) region, basic region, leucine zipper (ZIP) at the N-terminus, and transactivation domain containing proline-rich regions at the C-terminus. Stars indicate each proline-rich repeats. Red highlight the longer isoform found in CVI-988 with three more proline-rich repeat and three punctual mutations.

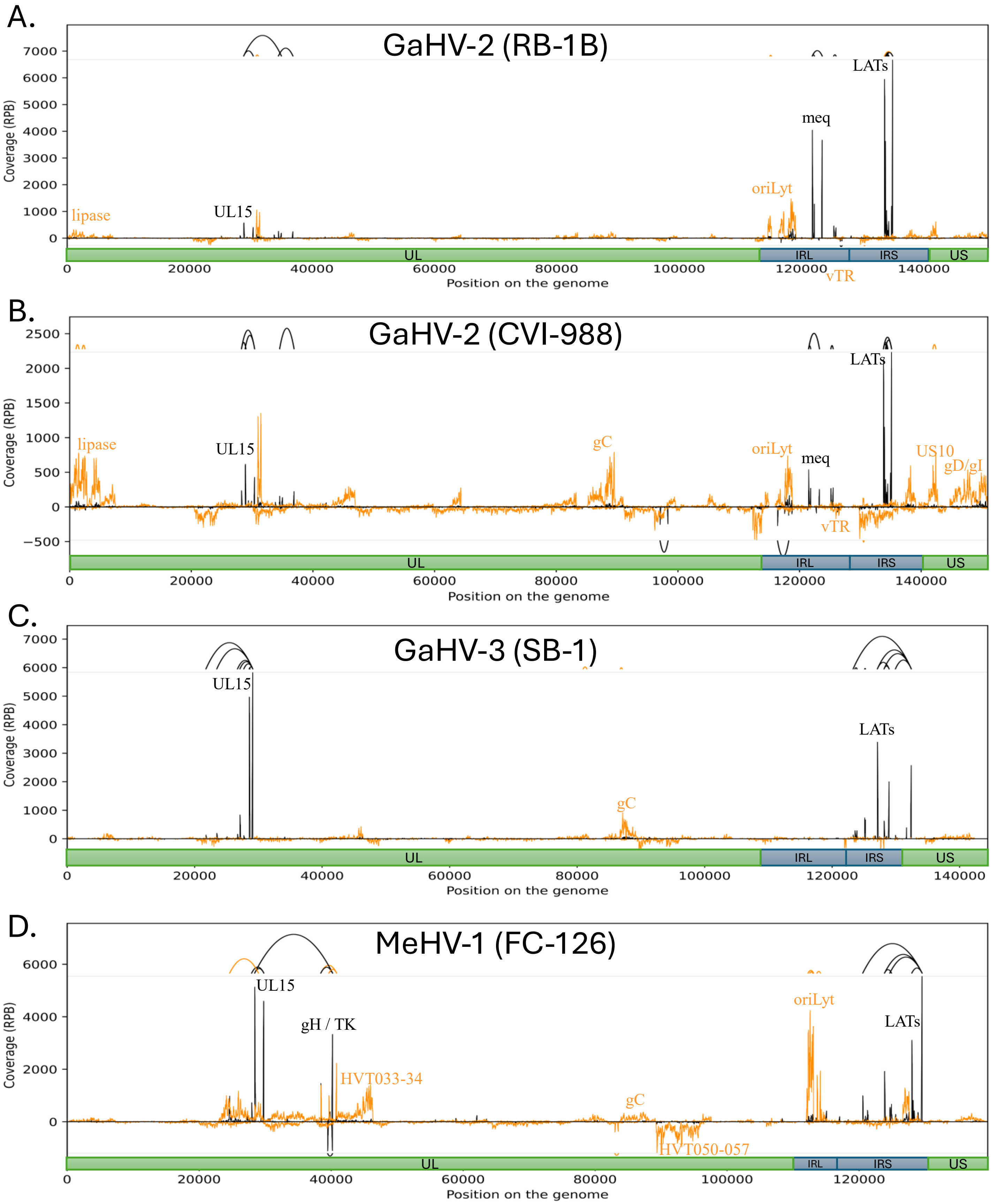

Figure S2 : **CircRNA coverage obtained after alignment on GaHV-3 (A), MeHV-1 (B) or GaHV-2 CVI988 (C).** The orange color depicts the coverage due to non-canonical circRNAs (U2-independent using splice sites different from (GU-AG)). The black color depicts the coverage due to canonical circRNAs. The X axis represents the genome. The Y axis represents the normalized coverage (circRNA reads per billion reads mapped on the viral genome), negative values indicate a mapping on the antisense strand of the viral genome. The arches above and below the graph represent the top-20 of most expressed circRNAs. The size of these arches is dependent on the circRNA expected size. Unique long (UL) and unique short (US) regions are depicted in green. Internal repeat long (IRL) and internal repeat short (IRS) regions are depicted in blue. External repeats were removed from the genome for convenience in the analysis.

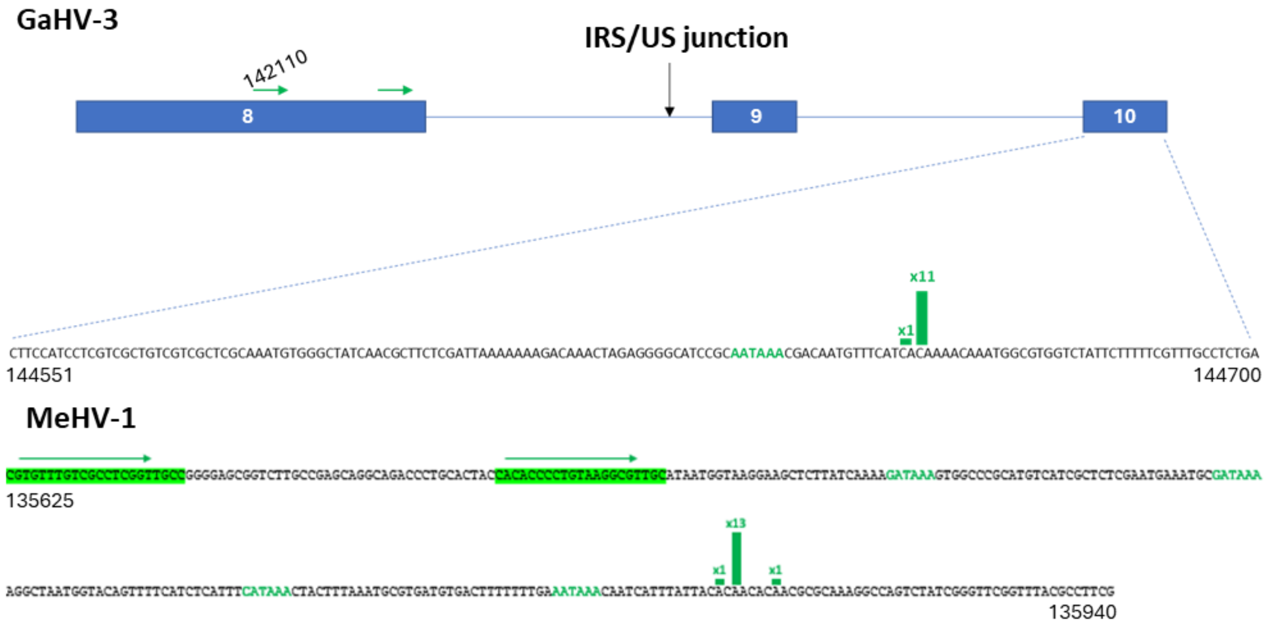

Figure S3 : **3' RACE PCR results showing Latency Associated Transcripts 3' ends in GaHV-3 and MeHV-1.** Blue boxes present the last exons of LATs. Green arrows highlight primers used for amplification. Green nucleotides identify polyadenylation signal. Positions on the corresponding genome are shown.

A

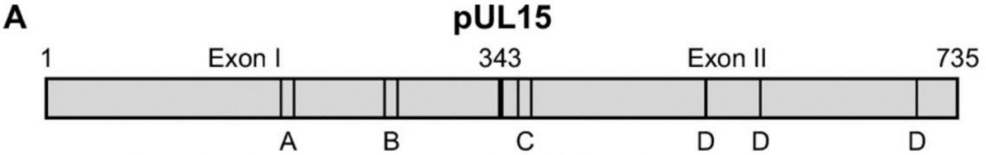

A. Nuclear localization signal: 183- PKKRAKV -189
B. Walker A box: 258- VPRRHGKT -265
C. Walker B box: 352- LLFVDE -357
D. Mn^{2+}-coordination: D509, E581, D707

B

```
808 bits(2086)  0.0     Compositional matrix adjust.  405/743(55%)  522/743(70%)  20/743(2%)

GaHV-2  1    MFGGLLGEETKRHFERLMKTKNDRLGASHRNERSIRDGDMVDAPFLNFAIPVPRRHQTVM  60
             MFG  L  + +++ ERL K +  ++GA   +      GD +  PFL+FA   P+RHQTV+
HHV-1   1    MFGQQLASDVQQYLERLEKQRQLKVGADEASAGLTMGGDALRVPFLDFATATPKRHQTVV  60

Query   61   PAIGILHNCCDSLGIYSAITTRMLYSSIACSEFD--ELRRDSVPRCYPRITNAQAFLSPM  118
             P +G LH+CC+   ++SA+  R+L++S+  ++    +   D   +          FL+P
Sbjct   61   PGVGTLHDCCEHSPLFSAVARRLLFNSLVPAQLKGRDFGGDHTAKL--------EFLAPE  112

Query   119  MMRVANSIIFQEYDEMECAAHRNAYYSTMNSFISMRTSDAFKQLTVFISRFSKLLIASFR  178
             ++R    + F+E    +    RNAYYS +N+F ++  S+AF+QL  F+  F++LL  SFR
Sbjct   113  LVRAVARLRFKECAPADVVPQRNAYYSVLNTFQALHRSEAFRQLVHFVRDFAQLLKTSFR  172

Query   179  DVNKLDDHT--VKKRARIDAPSYDKLHGTLELFQKMIFDACHLFCNFCFTWRSRRASERL  236
               + L + T   KKRA++D  ++ + +GTLELFQKMI      F
Sbjct   173  -ASSLTETTGPPKKRAKVDVATHGRTYGTLELFQKMILMHATYFLAAVLLGDHAEQVNTF  231

Query   237  LRVAFDTPHFSDIVTRHFRQRATVFLVPRRHGKTWFLVPLIALAMSSFEGIRIGYISHIR  296
             LR+ F+ P FSD   RHFRQRATVFLVPRRHGKTWFLVPLIAL+++SF GI+IGY +HIR
Sbjct   232  LRLVFEIPLFSDAAVRHFRQRATVFLVPRRHGKTWFLVPLIALSLASFRGIKIGYTAHIR  291

Query   297  KAIEPVFEDIGDRLRRWFGAHRVDHVKGETITFSFPSGLKSTVTFASSHNTNSIRGQDFN  356
             KA EPVFE+I   LR WFG+ RVDHVKGETI+FSFP G +ST+ FASSHNTN IRGQDFN
Sbjct   292  KATEPVFEEIDACLRGWFGSARVDHVKGETISFSFPDGSRSTIVFASSHNTNGIRGQDFN  351

Query   357  LLFVDEANFIRPDAVQTIIGFLNQATCKIIFVSSTNSGKASTSFLYGLKGSADDLLNVVT  416
             LLFVDEANFIRPDAVQTI+GFLNQA CKIIFVSSTN+GKASTSFLY L+G+AD+LLNVVT
Sbjct   352  LLFVDEANFIRPDAVQTIMGFLNQANCKIIFVSSTNTGKASTSFLYNLRGAADELLNVVT  411

Query   417  YICDEHMKHVTDYTNATSCSCYVLNKPVFITMDGAMRRTAEMFLPDSFMQEIIGGGVVDR  476
             YICD+HM  V  +TNAT+CSCY+LNKPVFITMDGA+RRTA++FL DSFMQEIIGG   +
Sbjct   412  YICDDHMPRVVTHTNATACSCYILNKPVFITMDGAVRRTADLFLADSFMQEIIGGQARE-  470

Query   477  TICQGDRSIFTASAIDRFLIYRPSTVNNQDPFSQDLYVYVDPAFTANTKASGTGVAVIGK  536
                  DR + T SA +RFL+YRPST  N    + DLYVYVDPAFTANT+ASGTGVAV+G+
Sbjct   471  --TGDDRPVLTKSAGERFLLYRPSTTTNSGLMAPDLYVYVDPAFTANTRASGTGVAVVGR  528

Query   537  YGTDYIVFGLEHYFLRALTGESSGSIGYCVAQCLIQICAIHRKRFGVIKIAIEGNSNQDS  596
             Y  DYI+F LEH+FLRALTG +   I  CV   L Q+ A+H   F  +++A+EGNS+QDS
Sbjct   529  YRDDYIIFALEHFFLRALTGSAPADIARCVVHSLTQVLALHPGAFRGVRVAVEGNSSQDS  588

Query   597  AVAIATRIAIEMISYM--KAAVAPTPHNVSFYHSKSNGTDVEYPYFLLQRQKTTAFDFFI  654
             AVAIAT +  EM   +  + A A +   + FYH +  G+ V YP+FLL +QKT AF+ FI
Sbjct   589  AVAIATHVHTEMHRLLASEGADAGSGPELLFYHCEPPGSAVLYPFFLLNKQKTPAFEHFI  648

Query   655  AQFNSGRVLASQDLVSTTVSLTTDPVEYLTKQLTNISEVVTGPTCTRTFSGKK-GGNDDT  713
              +FNSG V+ASQ++VS TV L TDPVEYL +QL N++E V+  T  RT+SGK+ G +DD
Sbjct   649  KKFNSGGVMASQEIVSATVRLQTDPVEYLLEQLNNLTETVSPNTDVRTYSGKRNGASDDL  708

Query   714  VVALTMAVYISAHI-PDMAFAPI  735
             +VA+ MA+Y++A   P   FAPI
Sbjct   709  MVAVIMAIYLAAQAGPPHTFAPI  731
```

Figure S4 : **Comparison of UL15 protein between HHV-1 and GaHV2: HHV-1's UL15 protein with its functional elements (A) (Heming *et al.*, 2017) and protein sequence alignment of the two viruses (B).** Sequence alignment was realized on BlastP with GaHV-2 sequence as query. Nuclear localization signal and Walker boxes are highlighted in yellow, the START methionine of the alternative protein in blue and Mn^{2+} important amino acids in green.
